# Prospective compliance assessment of surgical site infection prevention measures in colorectal surgery

**DOI:** 10.1093/bjsopen/zrad013

**Published:** 2023-04-03

**Authors:** Philip Deslarzes, Jonas Jurt, Martin Hübner, Dieter Hahnloser, Daniel Clerc, Laurence Senn, Nicolas Demartines, Fabian Grass

**Affiliations:** Department of Visceral Surgery, Lausanne University Hospital, Centre Hospitalier Universitaire Vaudois, University of Lausanne (UNIL), Lausanne, Switzerland; Department of Visceral Surgery, Lausanne University Hospital, Centre Hospitalier Universitaire Vaudois, University of Lausanne (UNIL), Lausanne, Switzerland; Department of Visceral Surgery, Lausanne University Hospital, Centre Hospitalier Universitaire Vaudois, University of Lausanne (UNIL), Lausanne, Switzerland; Department of Visceral Surgery, Lausanne University Hospital, Centre Hospitalier Universitaire Vaudois, University of Lausanne (UNIL), Lausanne, Switzerland; Department of Visceral Surgery, Lausanne University Hospital, Centre Hospitalier Universitaire Vaudois, University of Lausanne (UNIL), Lausanne, Switzerland; Infection Prevention and Control Unit, Department of Infectious Diseases, Lausanne University Hospital, Centre Hospitalier Universitaire Vaudois, University of Lausanne (UNIL), Lausanne, Switzerland; Department of Visceral Surgery, Lausanne University Hospital, Centre Hospitalier Universitaire Vaudois, University of Lausanne (UNIL), Lausanne, Switzerland; Department of Visceral Surgery, Lausanne University Hospital, Centre Hospitalier Universitaire Vaudois, University of Lausanne (UNIL), Lausanne, Switzerland

*Dear Editor*

Surgical site infections (SSIs) are associated with increased morbidity, and mortality rates, and increased healthcare costs^1^. There is increased implementation in healthcare of standardized care bundles to reduce SSIs. In the authors' institution, a multimodal SSI prevention bundle was introduced as the standard of care in November 2018 for colorectal resections and appendicectomies. The bundle consists of eight evidence-based items including antibiotic prophylaxis, skin disinfection, induction and perioperative core temperature control >36.5^°^C, intracavity lavage, selective abdominal drain placement, systematic use of a double-ring wound protection device, glove change before closure and predefined closure strategy^2^. While the SSI rate of 20 per cent could not be substantially decreased after colonic resections, a beneficial impact in patients undergoing appendicectomy was observed^2,3^. However, compliance with the standardized care bundle was a modest 77 per cent^2^. To identify areas for improvement, the present study aimed to assess the impact of challenging circumstances on bundle compliance.

This prospective observational study included consecutive patients who underwent elective or non-elective (surgery during a non-scheduled hospital stay) segmental or total colonic resections, rectal resections or appendicectomies between 1 November 2018 and 31 October 2020 (CER-VD # 2020e238 and CER-VD # 20162991). Compliance was assessed at the end of each procedure by the lead surgeon through dedicated checklists. To evaluate bundle compliance during challenging circumstances, three items were pragmatically chosen as surrogates for the complexity of the patient (age), the procedure (surgical duration) and the surgical setting (daytime *versus* nighttime).

In total, 1019 patients were included, of which 463 (45 per cent) underwent appendicectomy, 458 (45 per cent) colonic resection and 98 (10 per cent) rectal resection (*Table S1*). Compliance rates to intra-abdominal lavage, intra-abdominal drain placement and core temperature control standards decreased significantly with surgical duration of colonic resections (88 per cent, 86 per cent and 64 per cent for surgeries lasting <90 min *versus* 56 per cent, 68 per cent and 57 per cent for surgeries lasting >180 min respectively, all *P* < 0.050). Compliance to the predefined closure algorithm increased with surgical duration (62 per cent *versus* 88 per cent, *P* < 0.05). Compliance to antibiotic timing, wound protector and glove change recommendations was higher in patients undergoing daytime (08.00–16.00 h) *versus* night shift (24.00–08.00 h) colectomies (93 *versus* 69 per cent, 85 *versus* 72 per cent and 87 *versus* 58 per cent respectively, all *P* < 0.05). No significant difference in compliance rate was observed related to surgical timing in patients undergoing appendicectomy, and age had no impact on bundle compliance in both colonic and rectal resections (*Fig. 1*).

**Fig. 1:**
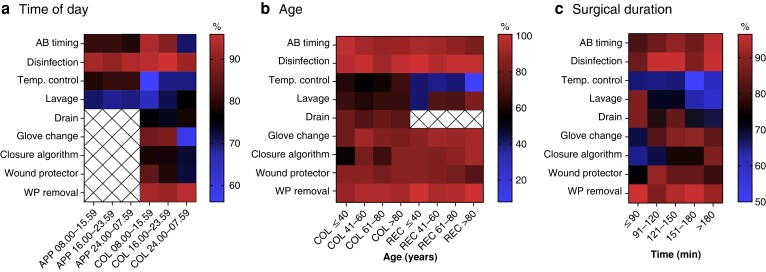
Heat map graph to reflect compliance patterns to individual bundle items **a** Related to time of day. **b** Related to age. **c** Related to surgical duration. AB, antibiotics; Temp., temperature; WP, wound protector; APP, appendicectomy; COL, colonic; REC, rectal. Colour codes display compliance in per cent according to the scale.

In summary, the present study revealed decreasing compliance during night shifts regarding antibiotic timing, use of wound protectors and glove changes. Several factors may contribute to these findings. Night-time operating was exclusively performed in an emergency setting and less senior clinical presence with an awareness of institutional protocol may have affected compliance. Higher intensity and variable workloads experienced during night shifts may also have reduced compliance with SSI prevention care bundles.

While compliance to intra-abdominal lavage standards, drain placement and core temperature control decreased significantly with long-lasting procedures, the predefined skin closure algorithm was implemented more often. Abdominal contamination needs immediate attention before contaminating clean areas; however, these principles may have been neglected with longer duration of surgery, and the tendency to lavage the entire abdomen after long and tiring procedures irrespective of contamination. The principle of limited lavage of contained contaminated areas prevents spillage and bacterial seeding of initially uncontaminated quadrants^4^.

There was approximately 60 per cent compliance to both lavage recommendations and temperature control, which is disappointingly low throughout the cohort. These are areas for quality improvement to reduce SSIs in our institution^5^. There are limitations to this study including its single centre methodology which may limit the external validity. However, many of these challenges, such as out of hours operating, are common to other international centres providing emergency surgical care.

## Supplementary Material

zrad013_Supplementary_Data

## Data Availability

Data are not made publicly available as they are part of an ongoing institutional quality improvement project.
