# Supplementary material for: Prospective compliance assessment of surgical site infection prevention measures in colorectal surgery
Source: BJS Open. 2023 Apr 3;7(2):zrad013. doi: 10.1093/bjsopen/zrad013 (PMC10069656; doi:10.1093/bjsopen/zrad013)
Supplement: zrad013_Supplementary_Data [file zrad013_supplementary_data.docx]

**Prospective compliance assessment to surgical site infection prevention measures in colorectal surgery**

Philip Deslarzes^a^, Jonas Jurt^a^, Martin Hübner^a^, Dieter Hahnloser^a^, Daniel Clerc^a^, Laurence Senn^b^, Nicolas Demartines^a^, Fabian Grass^a^

^a^ Department of Visceral Surgery, Lausanne University Hospital CHUV, University of Lausanne (UNIL), Switzerland

^b^ Infection Prevention and Control Unit, Department of Infectious Diseases, Lausanne University Hospital CHUV, University of Lausanne (UNIL), Switzerland

**Correspondence to:** PD Dr Fabian Grass

Department of Visceral Surgery

Lausanne University Hospital CHUV

Bugnon 46

1011 Lausanne

Switzerland

Phone: +41 21 314 65 60/ +41 79 556 88 38

E-mail : fabian.grass@chuv.ch

**Supplementary Materials - Index**

| **Supplementary Figures and Tables** |  |
| --- | --- |
| Table S1. Demographic and surgical characteristics | *pag. 2* |
|  |  |

|  | Appendicectomy  n=463 | Colonic resection  n=458 | Rectal resection n=98 |
| --- | --- | --- | --- |
| Age, median | 33 +/-16 | 66 +/-16 | 61 +/-18 |
| ≤40 years (%) | 301 (65.0) | 39 (8.5) | 13 (13.3) |
| 41-60 years (%) | 112 (24.2) | 127 (27.7) | 30 (30.6) |
| 61-80 years (%) | 44 (9.5) | 214 (46.7) | 44 (44.9) |
| >80 years (%) | 6 (1.3) | 78 (17.1) | 11 (11.2) |
| Male gender(%) | 259 (55.8) | 250 (54.6) | 56 (57.1) |
| ASA class >3 (%) | 37 (8.0) | 244 (53.3) | 32 (32.7) |
| BMI (Kg/m2, mean +/-SD) | 24.6 +/-4.9 | 26.2 +/- 5.7 | 25.5 +/- 5.1 |
| Emergency (%) | 450 (97.0) | 242 (52.8) | 8 (8.2) |
| Laparoscopy (%) | 457 (98.5) | 183 (40.0) | 61 (62.2) |
| Converted (%) | 3 (0.6) | 43 (9.4) | 15 (15.3) |
| Surgical duration (min, mean +/-SD) | 66 +/- 34 | 186 +/-106 | 253 +/- 113 |
| ≤90 minutes (%) | 404 (87.3) | 51 (11.1) | 1 (1.0) |
| 91-120 minutes (%) | 45 (9.7) | 74 (16.2) | 2 (2.1) |
| 121-150 minutes (%) | 6 (1.3) | 81 (17.7) | 7 (7.1) |
| 151-180 minutes (%) | 5 (1.1) | 59 (12.9) | 6 (6.1) |
| >180 minutes (%) | 3 (0.6) | 193 (42.1) | 82 (83.7) |
| 8am-1559pm (%) | 137 (29.6) | 339 (74.0) | 98 (100) |
| 1601 – 23.59 (%) | 123 (26.6) | 73 (15.9) | 0 (0) |
| 00am – 7.59am (%) | 203 (43.8) | 46 (10.1) | 0 (0) |

**Table S1.** Demographic and surgical characteristics.

ASA: American Standards Association, BMI: Body Mass Index. SD: Standard Deviation
